# Supplementary material for: Neuroimmune characterization of optineurin insufficiency mouse model during ageing
Source: Sci Rep. 2023 Jul 22;13:11840. doi: 10.1038/s41598-023-38875-3 (PMC10363168; doi:10.1038/s41598-023-38875-3)
Supplement: Supplementary file 1 — Supplementary Figures. [file 41598_2023_38875_MOESM1_ESM.pdf]

Supplementary Figure 1.

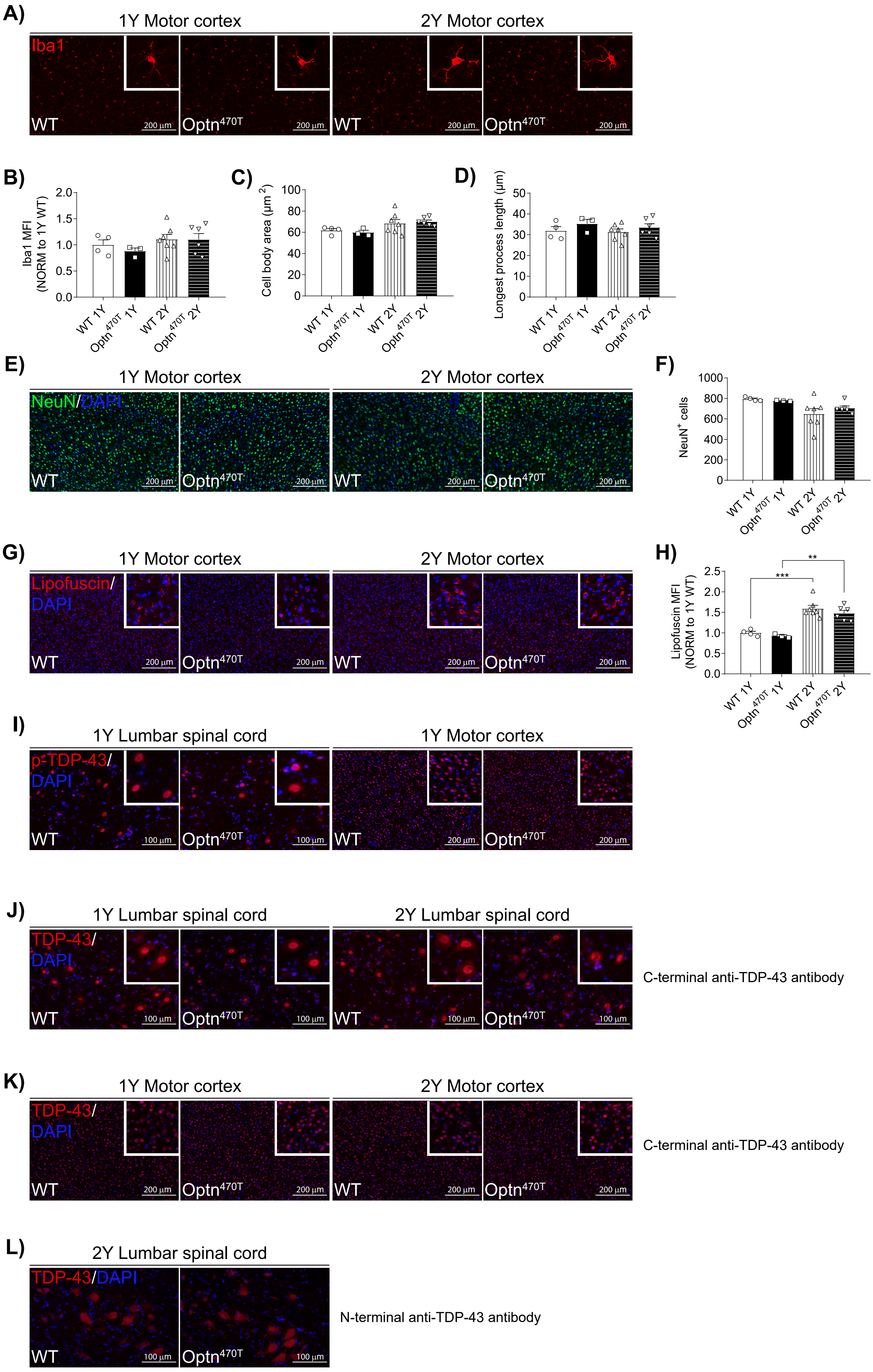

Supplementary Figure 2.

A)

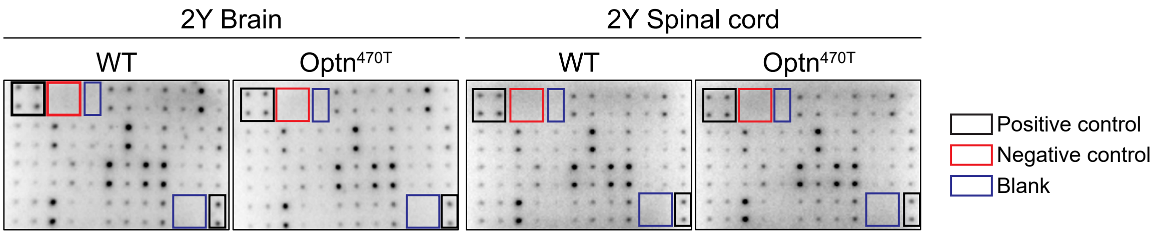

B) Brain

	CXCL13	CD30	CCL11	CCL24	FasL	Fractalkine	G-CSF
WT	1.2 ± 0.14	1.08 ± 0.16	0.6 ± 0.08	1.33 ± 0.20	0.93 ± 0.16	3.02 ± 0.28	0.28 ± 0.04
Optn^{470T}	1.18 ± 0.17	0.55 ± 0.19	0.41 ± 0.17	0.92 ± 0.12	0.66 ± 0.16	2.54 ± 0.20	0.28 ± 0.04

	GM-CSF	IFN-γ	IL-1α	IL-1β	IL-2	IL-3	IL-4	IL-6	IL-9	IL-10	IL-12	IL-12p70
WT	0.49 ± 0.02	0.51 ± 0.09	1.36 ± 0.15	0.08 ± 0.05	0.29 ± 0.05	0.3 ± 0.06	2.96 ± 0.33	0.34 ± 0.04	1.12 ± 0.13	0.29 ± 0.04	0.49 ± 0.05	0.96 ± 0.1
Optn^{470T}	0.46 ± 0.07	0.44 ± 0.08	1.44 ± 0.15	0.18 ± 0.01	0.44 ± 0.03	0.37 ± 0.05	2.72 ± 0.37	0.35 ± 0.08	0.91 ± 0.13	0.17 ± 0.07	0.51 ± 0.11	0.96 ± 0.09

	IL-13	IL-17A	I-TAC	CXCL1	Leptin	LIX	XCL1	CCL2	M-CSF	CXCL9	CCL3	MIP-1γ
WT	0.32 ± 0.005	0.29 ± 0.05	0.08 ± 0.04	0.56 ± 0.06	0.14 ± 0.01	2.54 ± 0.32	0.87 ± 0.05	2.6 ± 0.24	2.81 ± 0.23	0 07 ± 0 04	0.33 ± 0.08	0.26 ± 0.12
Optn^{470T}	0.45 ± 0.05	0.39 ± 0.07	**0.34 ± 0.05***	0.74 ± 0.04	0.26 ± 0.04	2.58 ± 0.11	0.96 ± 0.09	2.44 ± 0.29	2.54 ± 0.38	0.17 ± 0.08	0.51 ± 0.05	0.37 ± 0.04

	CCL5	CXCL12	TCA-3	CCL25	TIMP-1	TIMP-2	TNF-α	TNF RI	TNF RII
WT	0.3 ± 0.02	0.66 ± 0.13	2.13 ± 0.43	/	/	0.48 ± 0.09	0.59 ± 0.07	1.02 ± 0.12	0.44 ± 0.18
Optn^{470T}	0.47 ± 0.16	0.9 ± 0.1	2.62 ± 0.19	/	/	0.65 ± 0.05	0.82 ± 0.09	1.23 ± 0.09	0.62 ± 0.14

C) Spinal cord

	CXCL13	CD30	CCL11	CCL24	FasL	Fractalkine	G-CSF
WT	1.5 ± 0.26	1.37 ± 0.22	/	1.38 ± 0.29	0.92 ± 0.27	1.86 ± 0.66	0.34 ± 0.18
Optn^{470T}	1.81 ± 0.32	1.18 ± 0.11	/	1.37 ± 0.15	0.69 ± 0.12	1.59 ± 0.19	0.33 ± 0.07

	GM-CSF	IFN-γ	IL-1α	IL-1β	IL-2	IL-3	IL-4	IL-6	IL-9	IL-10	IL-12	IL-12p70
WT	0.58 ± 0.06	0.63 ± 0.07	1.5 ± 0.15	0.86 ± 0.14	0.2 ± 0.10	0.21 ± 0.12	3.74 ± 0.56	/	0.84 ± 0.15	/	0.63 ± 0.11	1.07 ± 0.23
Optn^{470T}	0.6 ± 0.01	0.86 ± 0.1	2.00 ± 0.49	1.19 ± 0.30	0.43 ± 0.25	0.43 ± 0.20	3.93 ± 0.53	/	1.02 ± 0.15	/	0.69 ± 0.07	0.96 ± 0.1

	IL-13	IL-17A	I-TAC	CXCL1	Leptin	LIX	XCL1	CCL2	M-CSF	CXCL9	CCL3	MIP-1γ
WT	0.6 ± 0.06	0.5 ± 0.09	1.05 ± 0.16	0.89 ± 0.07	/	3.6 ± 0.53	0.98 ± 0.05	3.22 ± 0.38	3.6 ± 0.49	/	0.64 ± 0.06	0.54 ± 0.11
Optn^{470T}	0.69 ± 0.01	0.68 ± 0.17	1.32 ± 0.28	1.18 ± 0.29	/	3.96 ± 0.42	**1.32 ± 0.1***	3.33 ± 0.28	3.45 ± 0.40	/	0.71 ± 0.01	0.32 ± 0.03

	CCL5	CXCL12	TCA-3	CCL25	TIMP-1	TIMP-2	TNF-α	TNF RI	TNF RII
WT	0.91 ± 0.02	1.43 ± 0.25	4.1 ± 0.61	/	/	0.99 ± 0.24	1.27 ± 0.21	1.18 ± 0.19	2.14 ± 0.36
Optn^{470T}	1 ± 0.04	1.66 ± 0.24	4.35 ± 0.54	/	/	0.83 ± 0.07	1.02 ± 0.1	0.69 ± 0.1	1.39 ± 0.21

Supplementary Figure 3.

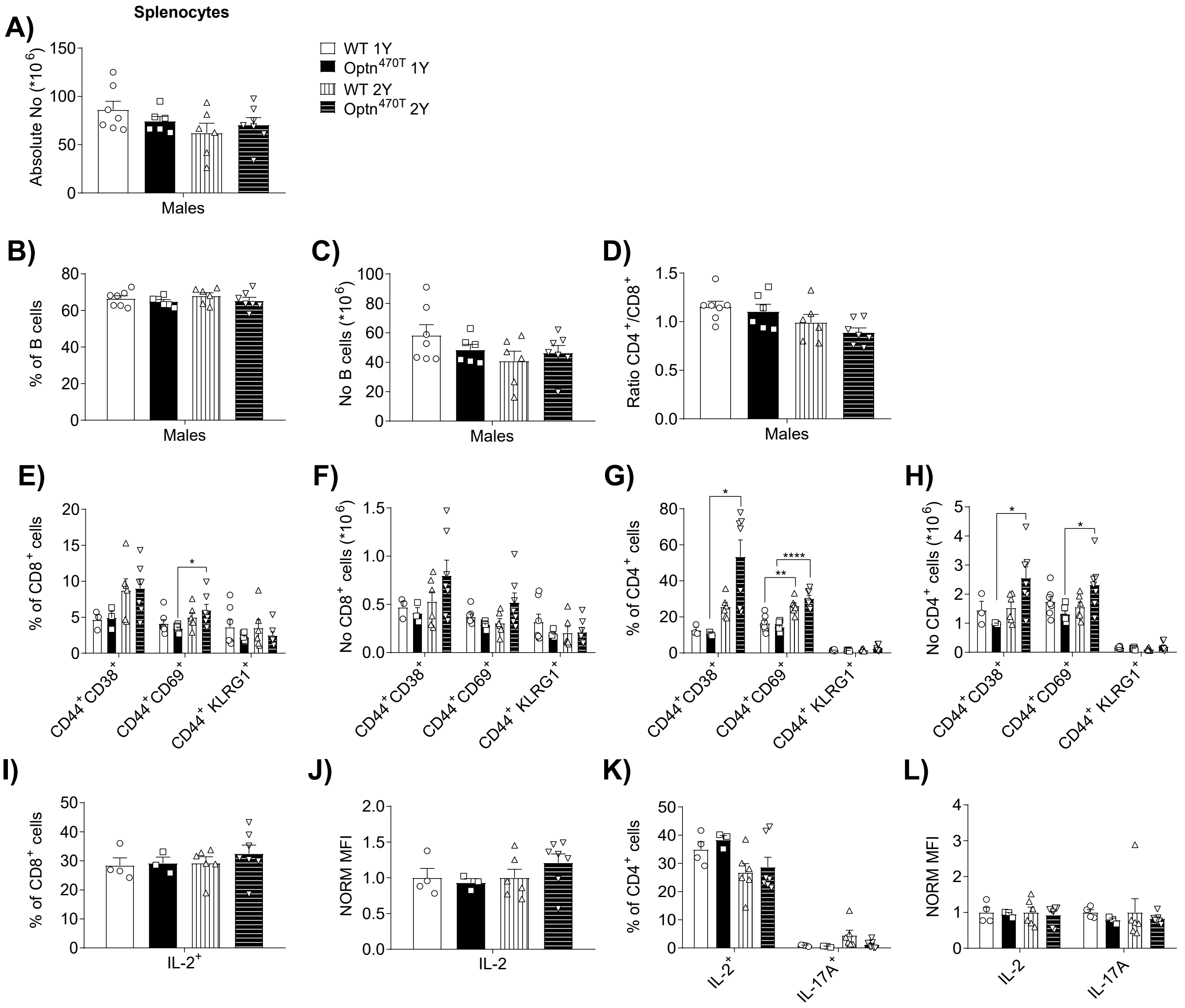

Supplementary Figure 4.

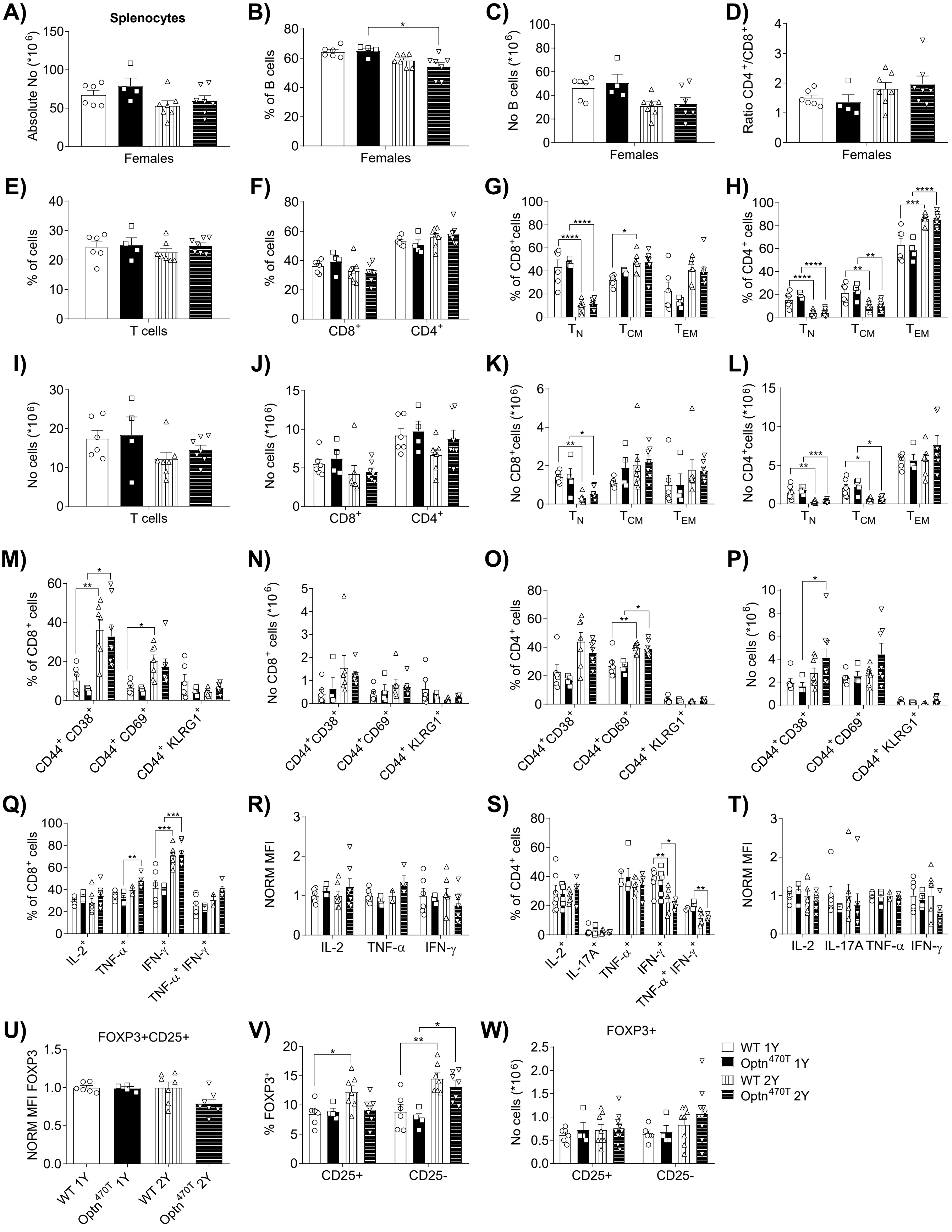

Supplementary Figure 5.

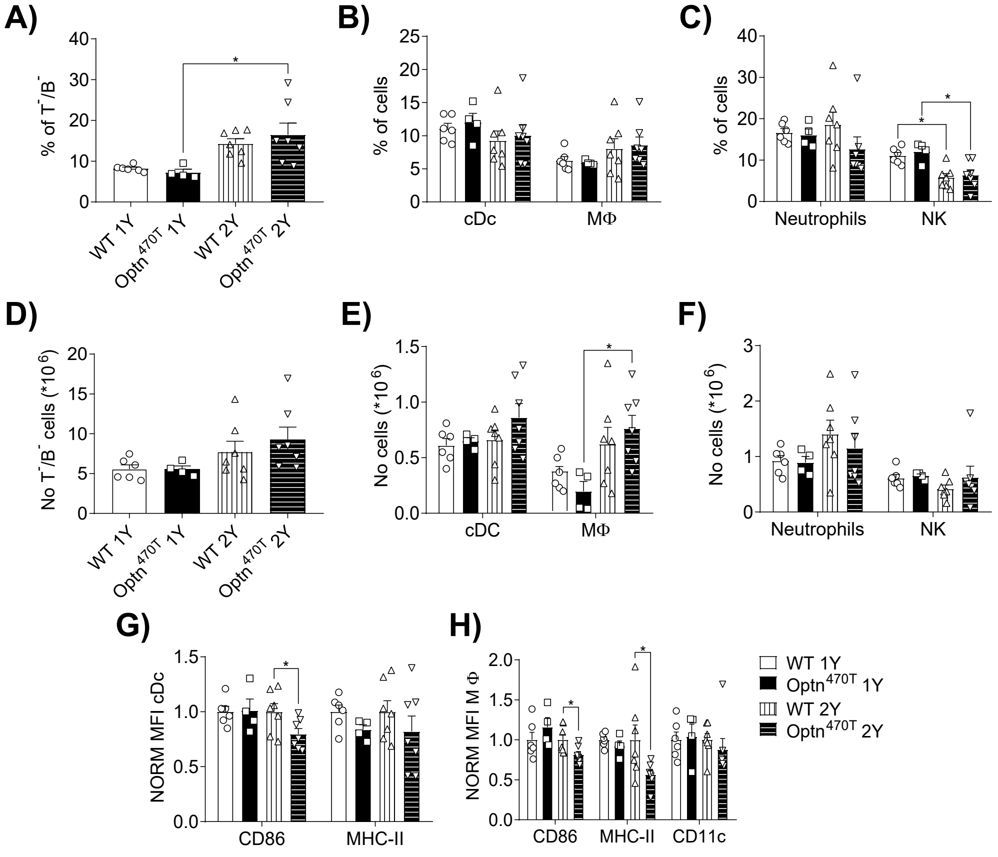

Supplementary Figure 6.

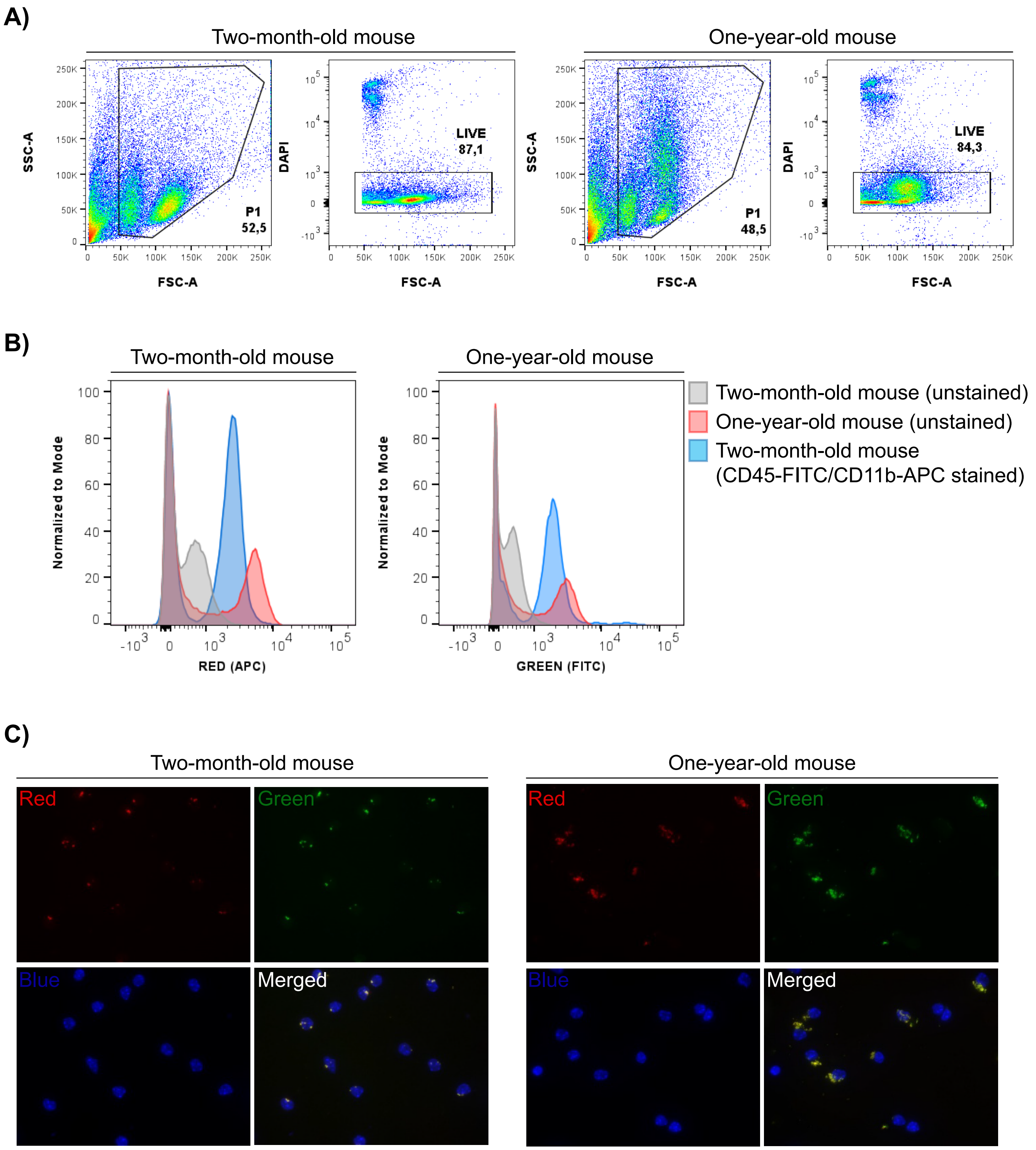

Supplementary Figure 7.

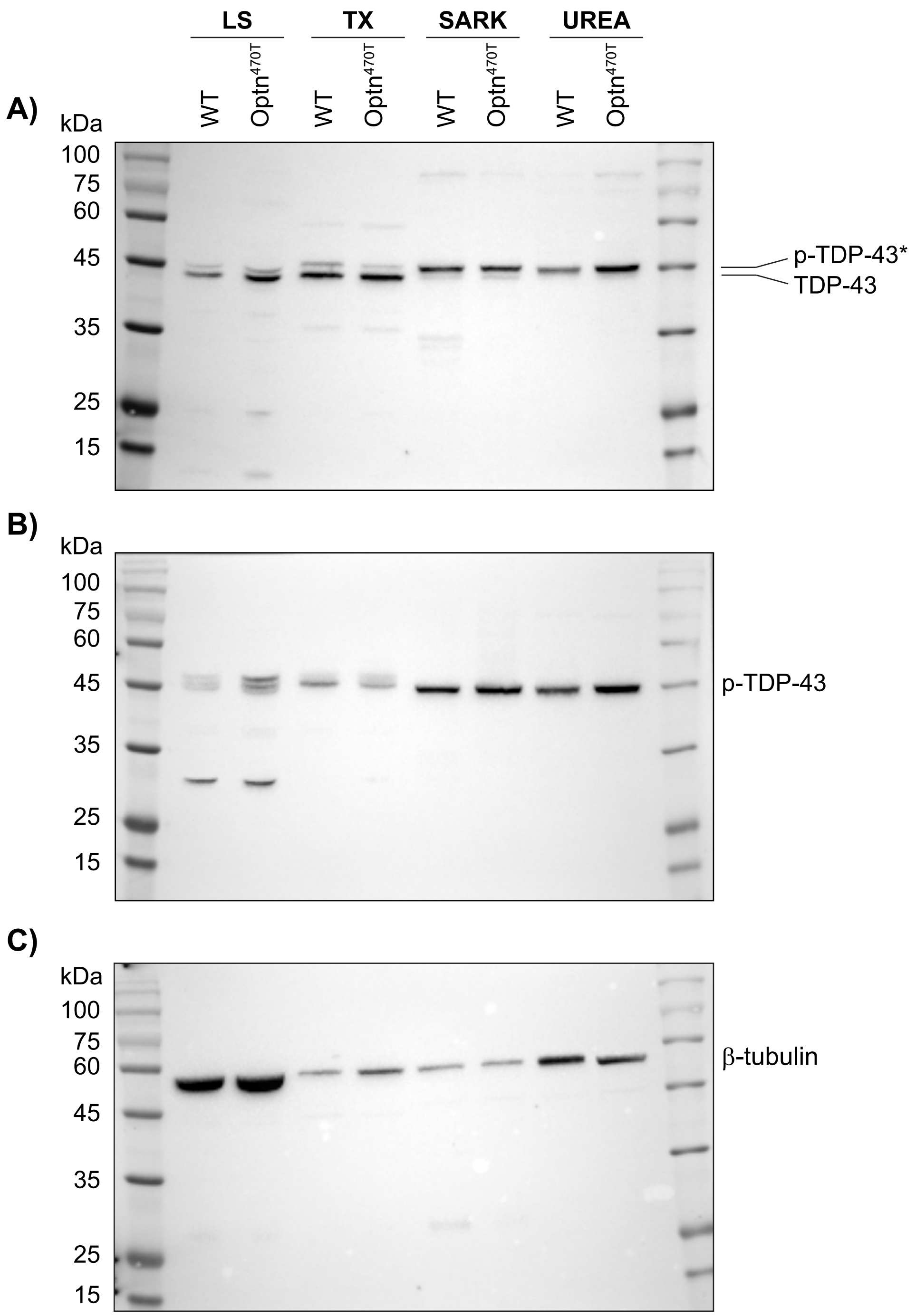

Supplementary Figure 8.

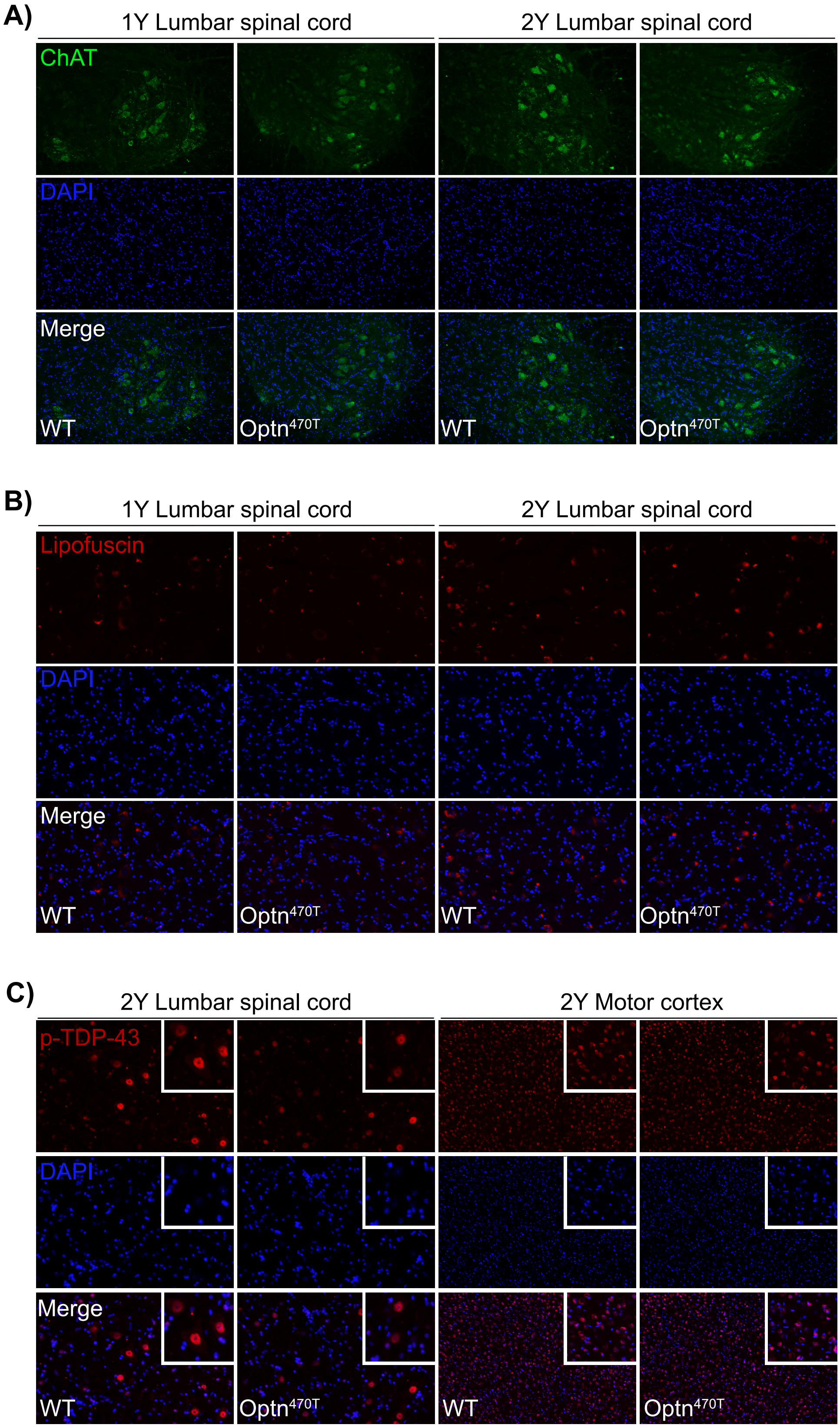

Supplementary Figure 8. (continued)

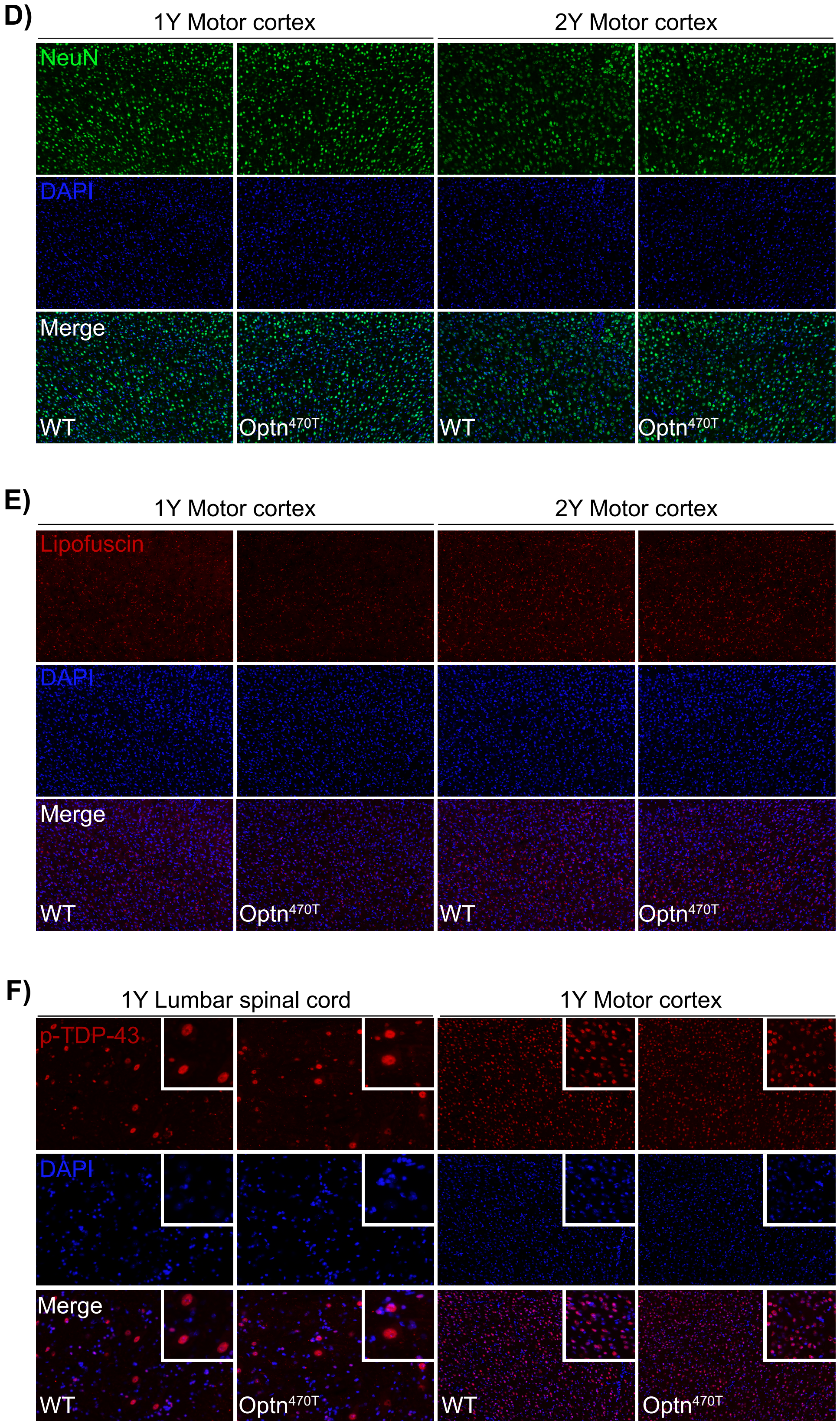

Supplementary Figure 8. (continued)

G)

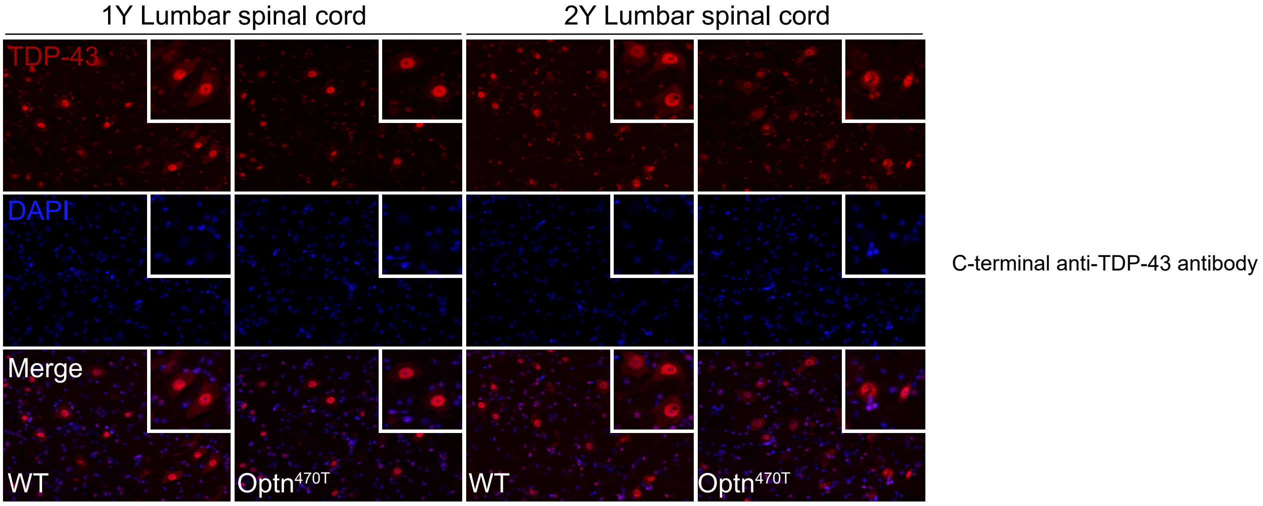

H)

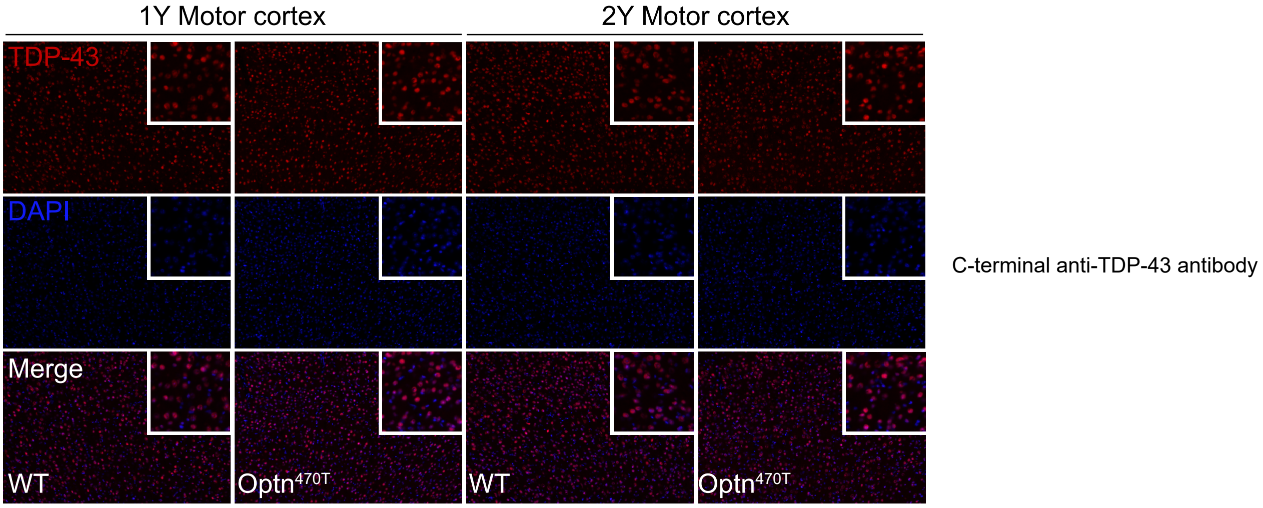

I)

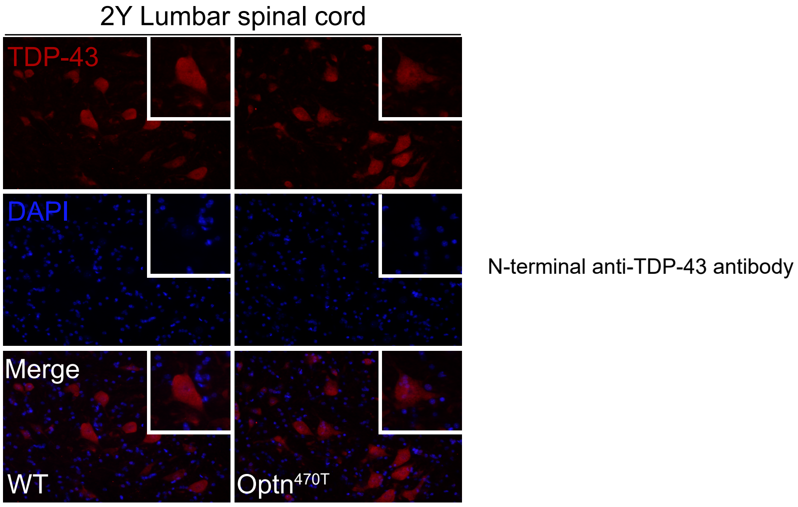

Supplementary Figure 1. Ageing did not induce ALS-like neuropathology in Optn^{470T} mice, continued. Motor cortex or lumbar spinal cord sections of one- and/or two-year-old mice were stained for Iba1 **(A)** and NeuN **(E),** phospho-TDP-43 **(I)**, C-terminal TDP-43 **(J-K)**, N-terminal TDP-43 **(L)** or were left unstained for lipofuscin visualization **(G)**; nuclei were identified by DAPI staining. MFI for Iba1 **(B)**, microglial cell body area **(C)**, longest process length **(D)**, NeuN$^+$ cell number **(F)**, and lipofuscin MFI **(H)** are shown. Data are presented as means ± SEM from 3-7 one-year- and two-year-old WT and Optn^{470T} mice and analysed by two-way ANOVA; **p<0.01, ***p<0.001. The scale bar is 100 μm (I, J, and L) and 200 μm (A, E, G, I, and K).

Supplementary Figure 2. Similar inflammatory profiles in the brains and spinal cords of aged Optn^{470T} and WT mice, continued. Representative RayBio® C-Series Mouse Inflammation Antibody Array C1 membranes incubated with brain lysates (left) and spinal cord lysates (right) from two-year-old male WT and Optn^{470T} mice are shown **(A)**. Mean values for individual proteins obtained by densitometric analyses ± SEM are shown for the brain **(B)** and spinal cord **(C)**. Three independent experiments were performed from the following groups of mice: 1) 4 WT and 5 Optn^{470T}; 2)1 WT, 5 Optn^{470T}; 3) 4 WT, 3 Optn^{470T}. The data were analysed by Student's *t*-test or Mann-Whitney test; * p<0.05.

Supplementary Figure 3. T cell subsets in aged Optn^{470T} were comparable to WT male mice, continued. Absolute splenocyte numbers from one- and two-year-old male WT and Optn^{470T} mice are shown **(A)**. Population frequency (%) **(B)** and absolute numbers (No) **(C)** of B cells, and CD4$^+$/CD8$^+$ ratio **(D)** are shown. Spleens of one- and two-year-old WT and Optn^{470T} male mice were stained for T cell activation markers, and gated as CD44$^+$CD38$^+$, CD44$^+$CD69$^+$, and CD44$^+$KLRG1$^+$ populations. CD8$^+$ **(E-F)** and CD4$^+$ **(G-H)** frequencies and numbers are shown. Staining for the indicated cytokines upon PMA/ionomycin stimulation is shown as frequency and MFI in CD8$^+$ cells **(I-J)** and CD4$^+$ cells **(K-L)**. The data from 6-7 one-year- and two-year-old WT and Optn^{470T} mice are shown as means ± SEM and analysed by two-way ANOVA (A-D, E: CD38; F: CD38 and CD69; G: CD69; H: CD38 and CD69; I, K: IL-2), Kruskal-Wallis test (E: CD69 and KLRG1; F: KLRG1; G: CD38 and KLRG1; H: KLRG1; and K: IL-17A), Student's *t*-test (J) and Mann-Whitney test (L); * p<0.05, ** p<0.01, ****p<0.0001.

Supplementary Figure 4. T cell subsets in aged Optn^{470T} were comparable to WT female mice. Absolute splenocyte numbers from one- and two-year-old female WT and Optn^{470T} mice are shown **(A)**. Population frequency (%) and absolute number (No) of B cells **(B-C)**, T cells **(E and I)**, $CD8^+$ and $CD4^+$ **(F and J),** and $CD4^+/CD8^+$ ratio **(D)** are shown. Frequencies and numbers for $CD8^+$ naïve (T_N), central memory (T_{CM}), and effector memory (T_{EM}) **(G and K)**, and $CD4^+$ T_N, $T_{CM,}$ and T_{EM} **(H and L)**. Spleens of one- and two-year-old WT and Optn^{470T} females were stained for T cell activation markers, and gated as $CD44^+CD38^+$, $CD44^+CD69^+$, and $CD44^+KLRG1^+$ populations. $CD8^+$ **(M-N)** and $CD4^+$ **(O-P)** frequencies and numbers are shown. Staining for the indicated cytokines upon PMA/ionomycin stimulation is shown as frequencies and MFI for $CD8^+$ **(Q-R)** and $CD4^+$ **(S-T)** cells. The graph shows the MFI of FOXP3 in Optn^{470T} normalized to one- and two-year-old WT female mice in $CD4^+FOXP3^+CD25^+$ Tregs **(U)**. The graphs show $CD25^+$ and $CD25^-$ Tregs as frequencies **(V)** and numbers **(W)**. The data from 4-8 one-year- and two-year-old WT and Optn^{470T} mice are shown as means ± SEM and analysed by two-way ANOVA (A, B, D, E, F, G: TN; H, I, J: CD4; K: TN; L: TN, TCM; M: CD38 and KLRG1; N: KLRG1; O: CD69 and KLRG1; QS; IL-2, IFN-γ and TNF IFN-γ; U, V), Kruskal-Wallis test (C, G: TCM and TEM; J: CD8; K: TCM and TEM; L: TEM; M: CD69; N: CD38 and CD69; O: CD38; P, S: IL-17A and TNF-a, W), Student's *t*-test (R, T: IL-2, TNF-a and IFN-g) and Mann-Whitney test (T: IL-17A); * $p<0.05$, ** $p<0.01$, ***$p<0.001$, ****$p<0.0001$.

Supplementary Figure 5. Innate immune characterization of WT and Optn^{470T} female mice. Spleens of one- and two-year-old WT and Optn^{470T} female mice were stained for innate immune markers. Population frequency (%) and absolute cell numbers (No) are shown as following: frequency of non-T/non-B cells **(A)**, conventional dendritic cells (cDc) and macrophages (MΦ) **(B)**, and neutrophils and NK cells **(C)**; numbers for non-T/non-B **(D),** cDc and MΦ **(E)**, and neutrophils and NK cells **(F)**. The MFI for indicated activation markers normalized to one- and two-year-old WT and Optn^{470T} mice are shown for cDc **(G)**, and MΦ **(H)**. The data from 4-8 one-year- and two-year-old WT and Optn^{470T} mice are shown as means ± SEM and analysed by two-way ANOVA (A, B, C: NK; D, E and F; Neutrophils), Kruskal-Wallis test (C: Neutrophils and F: NK), Student's *t*-test (E, F: CD86 and MHC-II) and Mann-Whitney test (F: CD11c); * $p<0.05$.

Supplementary Figure 6. Immune cells isolated from the aged brains showed high autofluorescence. Gating strategy for the immune cells isolated from the brains of two-month and one-year-old mice left unstained or stained for the indicated markers analysed by BD FACSAria^{TM} III cytometer **(A)**. Overlaid histograms show MFI from unstained and stained two-month-old, and unstained one-year-old immune cells in red (left) and green (right) channels **(B)**. Unstained immune cells isolated from two-month-old (left) and one-year-old (right) brains were seeded on coverslips, fixed, stained with DAPI, and analysed on Olympus IX83 fluorescent microscope **(C)**.

Supplementary Figure 7. Fractionation from individual mice. The brains of individual two-year-old WT and Optn470T mice were homogenized, biochemically fractionated, and immunoblotted for TDP-43 **(A)**, p-TDP-43 (pSer409) **(B)**, and β-tubulin (C); LS = low salt; TX = Triton X fraction; SARK = sarkosyl fraction.

Supplementary Figure 8. Single channel photomicrographs of lumbar spinal cord and motor cortex. Lumbar spinal cord and motor cortex sections from one- and two-year-old male mice were stained for ChAT **(A)**, phospho-TDP-43 Ser409 **(C** and **F),** NeuN **(D),** C-terminal TDP-43 **(G** and **H)** and N-terminal TDP-43 **(I)** or were left unstained for lipofuscin visualization **(B** and **E)**. Representative single channel photomicrographs from 6-7 WT and Optn^{470T} mice are shown.
